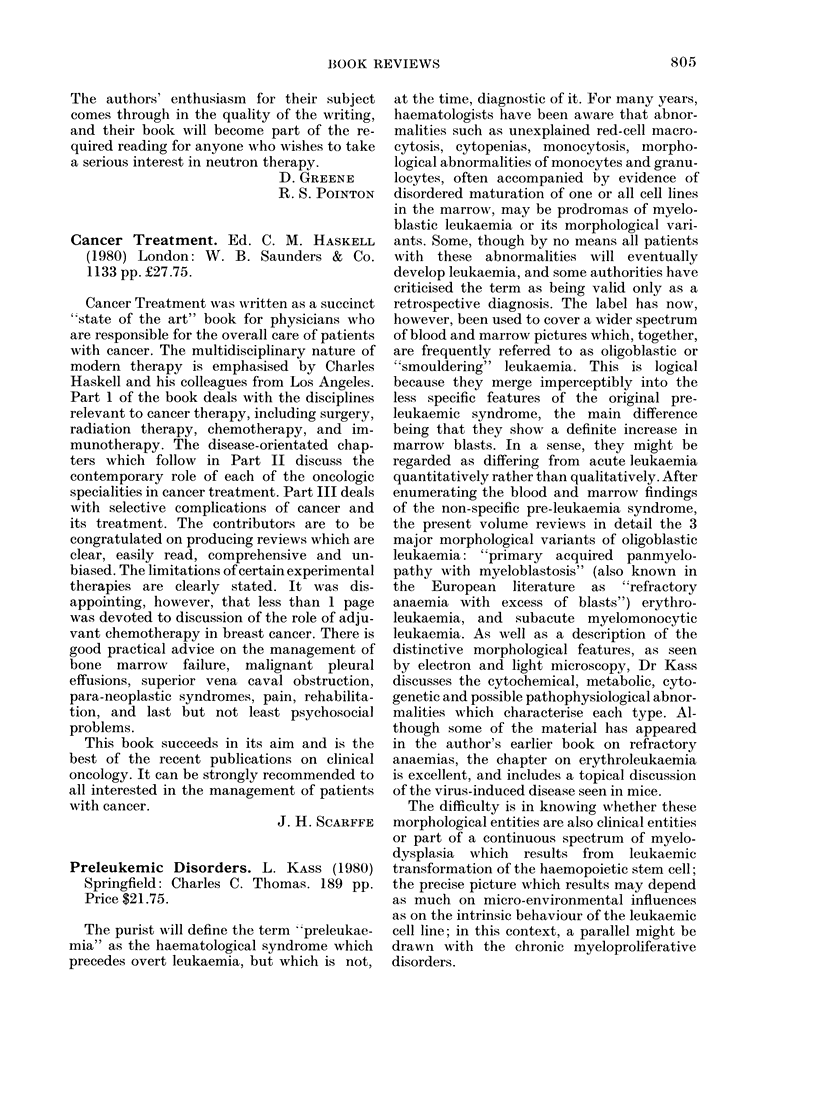# Cancer Treatment

**Published:** 1980-11

**Authors:** J. H. Scarffe


**Cancer Treatment.** Ed. C. M. HASKELL (1980) London: W. B. Saunders & Co. 1133 pp. £27.75.

Cancer Treatment was written as a succinct "state of the art" book for physicians who are responsible for the overall care of patients with cancer. The multidisciplinary nature of modern therapy is emphasised by Charles Haskell and his colleagues from Los Angeles. Part 1 of the book deals with the disciplines relevant to cancer therapy, including surgery, radiation therapy, chemotherapy, and immunotherapy. The disease-orientated chapters which follow in Part II discuss the contemporary role of each of the oncologic specialities in cancer treatment. Part III deals with selective complications of cancer and its treatment. The contributors are to be congratulated on producing reviews which are clear, easily read, comprehensive and unbiased. The limitations of certain experimental therapies are clearly stated. It was disappointing, however, that less than 1 page was devoted to discussion of the role of adjuvant chemotherapy in breast cancer. There is good practical advice on the management of bone marrow failure, malignant pleural effusions, superior vena caval obstruction, para-neoplastic syndromes, pain, rehabilitation, and last but not least psychosocial problems.

This book succeeds in its aim and is the best of the recent publications on clinical oncology. It can be strongly recommended to all interested in the management of patients with cancer.

J. H. SCARFFE